# Physiological Recordings of High and Low Output NMJs on the Crayfish Leg Extensor Muscle

**DOI:** 10.3791/2319

**Published:** 2010-11-17

**Authors:** Wen Hui Wu, Robin L. Cooper

**Affiliations:** Department of Biology, University of Kentucky

## Abstract

We explain in detail how to expose and conduct electrophysiological recordings of synaptic responses for high (phasic) and low (tonic) output motor neurons innervating the extensor muscle in the walking leg of a crayfish. Distinct differences are present in the physiology and morphology of the phasic and tonic nerve terminals. The tonic axon contains many more mitochondria, enabling it to take a vital stain more intensely than the phasic axon. The tonic terminals have varicosities, and the phasic terminal is filiform. The tonic terminals are low in synaptic efficacy but show dramatic facilitated responses. In contrast, the phasic terminals are high in quantal efficacy but show synaptic depression with high frequency stimulation. The quantal output is measured with a focal macropatch electrode placed directly over the visualized nerve terminals. Both phasic and tonic terminals innervate the same muscle fibers, which suggests that inherent differences in the neurons, rather than differential retrograde feedback from the muscle, account for the morphological and physiological differentiation.

**Figure:**
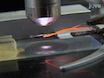


## Protocol

### 1) Introduction

Motor neurons communicate with a muscle fiber at synapses which are collectively referred to as a neuromuscular junction (NMJ). NMJs can be accessed easily in most crayfish muscle preparations. Many of the crayfish NMJs demonstrate non-spiking excitatory postsynaptic potentials (EPSP) similar to the graded electrical signals generated in postsynaptic dendrites within the mammalian CNS or subthreshold responses noted in vertebrate NMJs (Wiersma & Van Harreveld, 1938; Katz & Kuffler, 1946). The crayfish NMJs can serve as fundamental synaptic models to provide general insights into synaptic transmission and synaptic differentiation.

Generally, motor units regulate aspects of animal behavior via the type of synaptic communication at NMJs and properties of the muscles. Since the first observation of "fast" and "slow" muscle contractions in crab and crayfish closer muscle (Lucas, 1907, 1917), similar muscular contractile differentiation has been described in other crayfish muscle types such as abdominal flexors (Kennedy & Takeda,1965a, b) and limb extensors (Van Harreveld & Wiersma, 1936). "Fast" contractions initiate quick responses. For example, the crayfish tail flip is a fast behavior. "Slow" contractions maintain slow movements and help maintain posture (Bradacs *et al*., 1997). Corresponding to "fast" and "slow" muscle contractions, "phasic/high output" and "tonic/low output" are broadly used to describe the motor neurons. The difference in rate and timing of muscle contraction is in part related to presynaptic differences in synaptic structure and synaptic strength (King *et al*., 1996). Myofibrillar protein isoform expression is also important in contractile differences, but in preparations like that of the leg extensor muscle, in which a given fiber is innervated by both types of motor neurons, the focus is on synaptic differences of the terminals, since the terminals share the same target cell (Mykles *et al*., 2002). An earlier study examined the two excitatory motor axons of the leg extensor and described the phasic and tonic phenotypes (Bradacs *et al*., 1997). In this report, we demonstrate how to perform the dissection and obtain recordings so that others can further investigate the properties of the synaptic differentiation of these nerve terminals.

Viewed with transmission electron microscopy, various series of sections obtained from the tonic and phasic terminals on crayfish leg extensor muscle revealed that tonic terminals contain more RRP vesicles than phasic terminals; the mitochondria are more prevalent in tonic neurons, and the synapses on phasic terminals are more complex than those on the low-output synapses, since they contain multiple active zones with varied spacing (Miller *et al*, 2002; Johnstone *et al*., 2008; King *et al*., 1996; Bradacs *et al*., 1997). The low-output tonic terminals are also more susceptible to enhancing synaptic transmission with the neuromodulator serotonin (5-HT) than are the phasic terminals (Cooper *et al*., 2003).

The fact that the tonic and phasic NMJ are present on the same muscle fiber makes it easier to assess presynaptic differences on a given muscle fiber and to address questions of muscle fatigue, synaptic depression and synaptic cross talk. Various questions remain to be addressed in this preparation, such as whether there are differences in the postsynaptic receptor density and glutamate receptor subtypes in postsynaptic targets for the tonic and phasic terminals, but a better understanding of the fundamental differences in the anatomy and physiology of these two motor units will aid in building a more profound knowledge base. The hope is that the fundamental principles learned in this synaptic preparation will be applicable to other synapses in various preparations and will enhance future investigations in this synaptic model of the crayfish.

### 2) Methods

1. All the experiments are conducted on the first or second walking legs of midsize crayfish (*Procambarus clarkii*). The animals are individually housed in plastic containers with oxygenized water. The temperature of the animal room is in the range of 13°C-16°C. The animals are fed with dry fish food and the water changed on a weekly basis. 
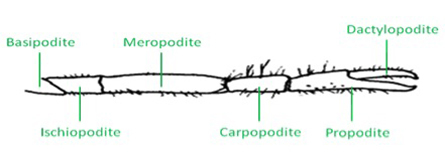
**Figure 1:** Schematic of a crayfish walking leg and the six distal segments.
2. The distal aspect of the walking leg in a crayfish is anatomically divided into six segments (Figure 1). The leg extensor is located in the meropodite, and the nerve bundle that will be isolated is close to the meropodite ischiopodite joint. The tonic or phasic axon can be selectively stimulated as needed for physiological purposes after they are exposed.
3. Cooper and Cooper (2009) described some aspects of the initial dissection, including methods for exposing the excitor of the opener motor neuron within the meropodite region, but their description does not provide the care required for protecting the extensor muscle from damage, as it was not needed to address the opener muscle preparation. In order to protect the extensor, the first or second walking leg is removed from the crayfish, measuring 6-10 cm in body length (Atchafalaya Biological Supply Co., Raceland, LA), by inducing the animal to automize the limb with forceful pinching distal to the fracture plane in the ischiopodite segment. The leg is placed on the dissection plate with the lateral (outer side) facing the viewer. The leg is turned around until the viewer can be sure the outside (lateral side) is facing up on the dissection plate, usually with the arched side up (Figure 2). Placing the leg on a piece of tissue paper makes it easier to turn the preparation while making these cuts. 
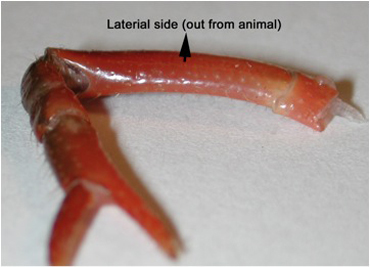
**Figure 2:** The lateral side of the meropodite, usually the side that is arched, is facing up on the dissection plate.
4. With a scalpel blade breaker and holder, a sharp razor blade is used to etch the cuticle until just cutting through in the pattern shown in Figure 3 for the meropodite segment. Care is taken not to cut too far distal on the dorsal to ventral cut by the meropodite-carpopodite joint. 
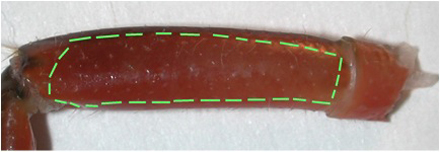
**Figure 3:** The meropodite segment with lines as a suggested pattern for etching out the window of cuticle.The preparation is placed in saline. The dissection dish should have a Sylgard (Dow Corning) coating on the bottom (1cm thick).The Sylgard is used so that insect pins can be stuck into it for holding the preparation still. At this point, a pin is stuck in the middle of the carpopodite segment and in the dorsal aspect of the ischiopodite segment (Figure 4). Dissected preparations are bathed in standard crayfish saline, modified from Van Harreveld s solution (1936), which is made with 205 NaCl; 5.3KCl; 13.5 CaCl_2_; 2H_2_O; 2.45 MgCl_2_; 6H_2_O; 5 HEPES and adjusted to pH 7.4 (in mM). 
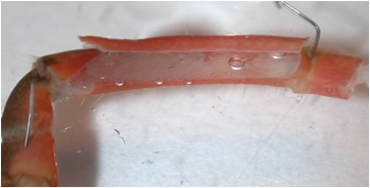
**Figure 4:** The meropodite segment with cut window being lifted off. Note the location of the dissection pins.
5. The cuticle is gently lifted in the distal region and the muscle fibers are cut away from the cuticle by making strokes towards the base of the leg. The cuticle can be lifted off.
6. The apodeme (tendon) is cut at the meropodite-carpopodite joint. A pin is placed on the inner surface of the flexor tendon to show where the cut is to be made (Figure 5). The tendon is then pinched where it was cut with tweezers and the flexor muscle pulled off by lifting it in a caudal direction (Figure 6), exposing the main leg nerve and the extensor muscle.
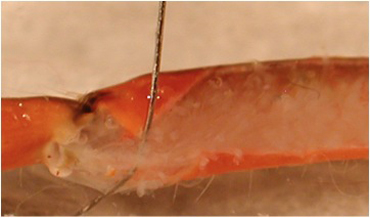
**Figure 5:** The apodeme of the flexor muscle is highlighted by displacing it from the flexor with a pin. 
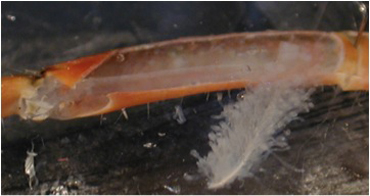
**Figure 6:** The apodeme of the flexor muscle is cut and removed with care as not to damage the main leg nerve.
7. The main leg nerve is cut at the meropodite-carpopodite joint and carefully pulled back over the extensor muscle. The medial surface of the muscle is used throughout this study. The separation of the nerve to the extensor muscle from the main leg nerve can be enhanced by gently pulling the distal stump of the main leg nerve to the side of the preparation. When peeling the main leg nerve back over the extensor muscle, small branches of axon may need to be cut. These are branches from the inhibitory motor neuron to the extensor muscle. The larger bundle branching off from the main leg nerve near the proximal end of the meropodite is the small nerve bundle of interest. 
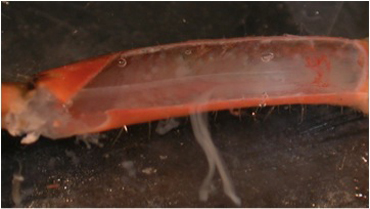
**Figure 7:** The main leg nerve is cut and pulled back in a proximal direction.This nerve bundle can be seen with methylene blue staining (Figure 8) or with 4-Di-2-ASP fluorescent stain (Figure 9). 
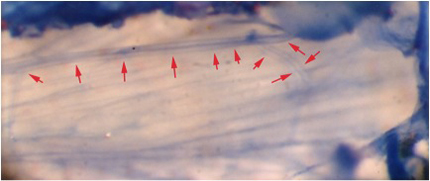
**Figure 8:** The extensor muscle stained with methylene blue. Note the axon branching and the two readily visible axons within the nerve. Red arrows demark the nerve track.
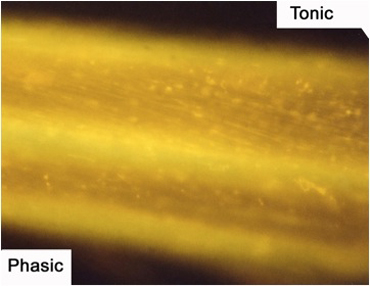
**Figure 9:** The axons of the motor nerve stained with 4-Di-2-ASP. The tonic axon is more brightly visible due to the increased mitochondrial content. 
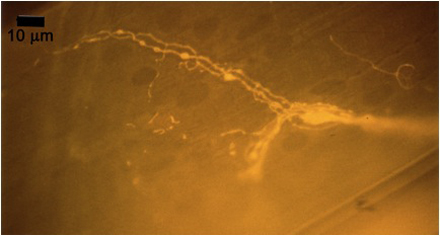
**Figure 10A:** Individual terminals of phasic and tonic neurons stained with 4-Di-2-ASP. Note the varicosities on the tonic terminals and the thin nature of the phasic terminals.
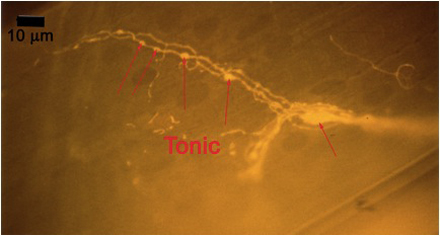
**Figure 10B** -Tonic noted
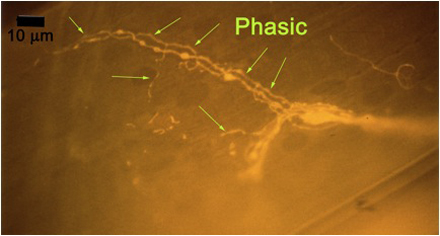
**Figure 10C** -Phasic noted

### 3) Physiological Profiles

1. To observe the excitatory postsynaptic potentials (EPSPs) of the tonic or phasic neurons, one of the isolated axons in the nerve bundle is stimulated by a suction electrode (Figure 11) connected to a Grass stimulator while intracellular potentials in the muscle are monitored (Johnstone *et al*., 2008). Stimulation at 70 Hz is applied to the tonic axon in order to promote a facilitated response for the low output NMJs, or a single pulse (1 Hz) is applied to the phasic axon in order to obtain large EPSPs of the high output NMJs as shown in Figure 12. The EPSPs are recorded to a computer via a PowerLab/4s interface.
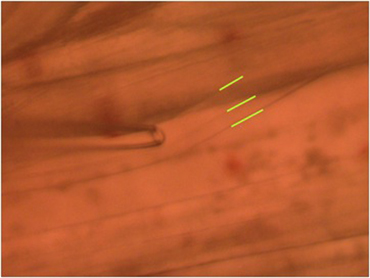
**Figure 11:** Stimulating electrode placed over a single axon within the nerve bundle. Note the green line outlining the 2 main axons.
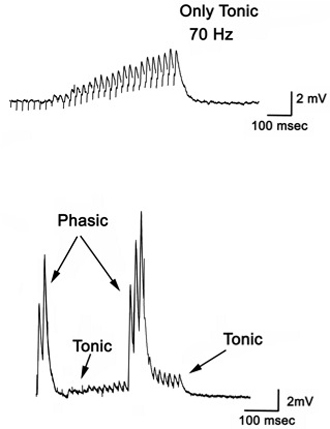
**Figure 12:** Postsynaptic potentials (EPSPs) of the tonic or phasic neurons as obtained by intracellular recordings.
2. Investigation in the nature of synaptic facilitation and synaptic depression can be approached by various experimental paradigms with the low and high output NMJs. Facilitation of the low output NMJs depends on the frequency, as shown for the 20, 40 and 60 Hz pulses of approximately 20 stimuli (Figure 13). 
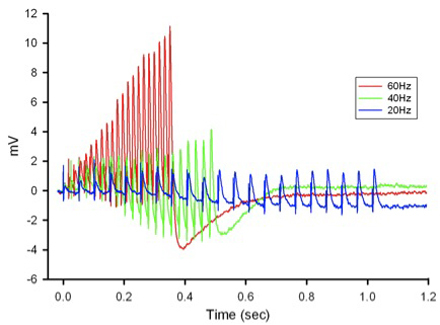
**Figure 13:** EPSPs in response to a train of stimulation pulses given at three different frequencies 20, 40 and 60 Hz in normal crayfish saline.
3. The rate of synaptic depression to frequency of stimulation is also related to the high output NMJs. Figure 14 shows a continuous 5 Hz of stimulation depressing the NMJ over 30 min. With higher stimulation frequency, the preparation will depress more rapidly (Bradacs *et al*., 1997).
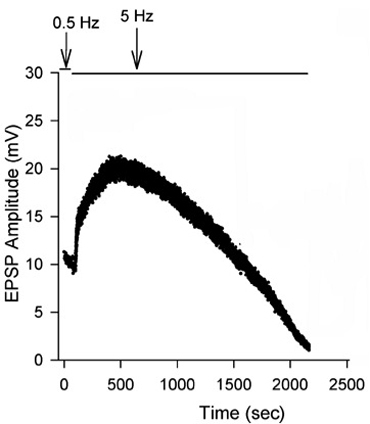
**Figure 14:** EPSP amplitude of the phasic response during 5 Hz stimulation to induce depression.

### 4) Quantal Responses

1. A procedure similar to that described for the opener muscle of the crayfish (Cooper and Cooper, 2009) is used in this preparation. The quantal EPSPs directly over identifiable regions of the nerve terminal are recorded by placing the lumen of a macro-patch recording electrode on synaptic varicosities visualized with the vital dye 4-Di-2-Asp (5 μM, 5-min treatment, Cooper *et al*., 1995; Magrassi *et al*., 1987). The spontaneous as well as evoked quantal responses can be recorded along the nerve terminals. The evoked and spontaneous synaptic potentials are recorded with the macro-patch electrode (Dudel, 1981; Wojtowicz *et al*.,1991; Mallart, 1993). Kimax glass (outer diameter: 1.5 mm) was pulled and fire-polished to produce patch tips with inside diameters ranging from 10 to 20 μm (Figure 15). 
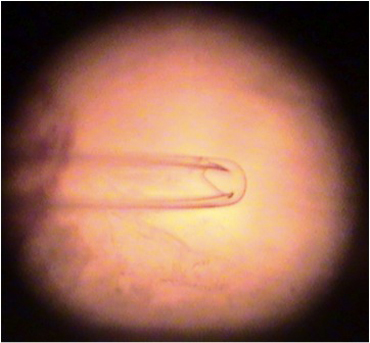
**Figure 15:** The lumen of a macro-patch recording electrode.The lumen of the electrode is filled with the bathing medium. The amplifier is the same as that used for the intracellular recordings mentioned above. Electrode and seal resistance can be determined by passing test current pulses through the electrode. In our experiments, seal resistances ranged from 0.3 to 1.0 MOhm and the electrode resistance ranged from 0.5 to 1.0 MOhm. Seal resistance can be monitored throughout the recording.
2. Direct counting of quantal events is possible with low stimulation frequencies. For each evoked response, the number of quantal events can be readily determined for the low output terminals (Figure 16). These direct counts can help estimate the mean quantal content (Del Castillo & Katz, 1954; Cooper *et al*., 1995). Since the evoked high output NMJs produce multi-quantal evoked events, the mean amplitude or area of the deflections, along with the average peak amplitude or area of the spontaneous events, can be used to approximate the mean quantal content (Cooper *et al*., 1995).
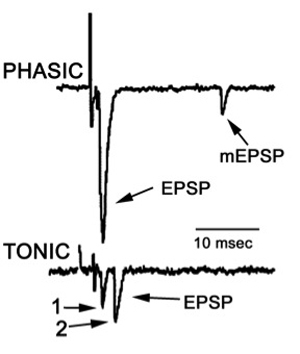
**Figure 16** Focal traces recorded from a phasic and a tonic NMJ.
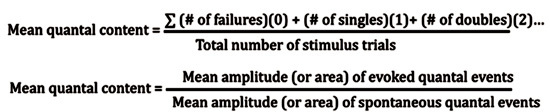
**Figure 17**

## Discussion

We have demonstrated in this report how to dissect, record and quantify synaptic responses in a unique crayfish neuromuscular preparation in which both high- and low-output terminals innervate the same muscle fiber. The neuromuscular preparations in the crayfish offer many advantages over vertebrate neuromuscular junctions, since only a few excitatory motor neurons are needed to innervate a muscle, and since the neurons are identifiable from preparation to preparation (Atwood, 1976). In addition, the excitatory neurotransmitter is glutamate, and the excitatory postsynaptic potentials (EPSP) are graded; thus, the biophysical properties of graded events are analogous to the dendrites of neurons within the CNS of vertebrates. The quantal currents, however, can be monitored directly at the postsynaptic sites (Cooper *et al*., 1995).

Repetitive 5 Hz stimulation of the phasic nerve gives rise to large EPSPs that become greatly depressed after several minutes. This type of depression is common in arthropod phasic neuromuscular junctions (Atwood and Cooper, 1996). The presence of the tonic terminals alongside the phasic terminals allows one to assess whether or not the postsynaptic target is greatly modified during and after depression of the phasic motor neuron. In addition, the low output terminals provide a nice preparation to investigate mechanisms that underlie synaptic facilitation (Desai-Shah *et al.,* 2008; Desai-Shah and Cooper, 2009)

The high output terminals provide a play ground to investigate modulation in the rate of synaptic depression and the recovery process. Accessible and viable preparations should help in deciphering the mechanisms behind synaptic depression. In this crayfish leg extensor preparation, exogenous application of serotonin is another tool to investigate recovery of synaptic depression and potentially a means of furthering investigation into the mobilization of synaptic vesicle pools. This preparation provides several experimental advantages, since individual muscle fibers are innervated by both phasic and tonic motor neurons. 

Since serotonin increases the number of vesicles that are released with evoked stimulation, and since it promotes recovery during depression, it is apparent that there is some modulation of the vesicle pools for enhancing the probability of fusion with serotonin present (Johnstone *et al.,* 2008; Logsdon *et al.,* 2006; Sparks *et al.,* 2004). Models that would explain the dynamics of the vesicle pools within the presynaptic nerve terminal during low frequency stimulation, as opposed to a depressed state, also need to be considered among the various experimental protocols that are possible with this preparation. 

It has been noted in other systems that about 30% of the vesicle pool undergoes a rapid recycling, whereas the rest of the recycled vesicles go through a traditional slow recycling path through the endoplasmic reticulum (Harata *et al.,* 2001; Tsien *et al.,* 2001). Such dual paths may also be present in this system. If ATP is lacking in the depressed state of the presynaptic terminal, then docking and undocking might not be able to occur; thus, many more unloaded vesicles would remain at the synaptic surface. Since more vesicles are rapidly released when the terminals are exposed to 5 HT, it is feasible that more are contained within the readily releasable pool (RRV). However, in the depressed state, with reduced ATP, the docking and undocking may be blocked even in the presence of 5 HT, which again leaves unloaded vesicles at the synaptic surface. Since the preliminary data suggests that more vesicles are released over time with prolonged 5 HT exposure and stimulation, the distribution of the vesicle pool from the fast and slow recycling paths may be skewed to have competent vesicles for re-release (from Johnstone *et al.,* 2008; see reviews- Desai-Shah *et al.,* 2008; Desai-Shah and Cooper, 2009)

With the techniques of focal macropatch recordings of postsynaptic currents and measures of single quanta from defined regions of the motor nerve terminal, one can ask questions to determine whether synaptic depression is occurring as a result of fewer vesicles being released or because of alterations of the function of postsynaptic receptors. It has been shown that the vesicles are more sensitive to fusion at the phasic NMJ of this preparation for an equal calcium exposure (Miller *et al.* 2005), which likely accounts for the higher mean quantal content of the phasic terminals (Msghina *et al.,* 1998, 1999). In addition, differences in the calcium binding protein frequenin (Jeromin *et al.,* 1999) and ultrastructure (King *et al.,* 1996) contribute to the differential synaptic efficacy (Cooper *et al.,* 2003).

Some prior studies have explored the muscle phenotype of the extensor muscle fibers (Bradacs *et al.,* 1997; Cooper *et al.,* 2003). Comparing the regulation of muscle differentiation for purely tonic and phasic fiber types in the crayfish to mixed fiber types, like for the leg extensor that is dully innervated, could provide clues to muscle phenotype expression and regulation (LaFramboise *et al.,* 2000; Sohn *et al.,* 2000; Griffis *et al.,* 2001; Mykles *et al.,* 2002).

Many fundamental questions remain to be addressed in neurobiology, and this preparation may aid in tackling some of them. A few topics of interest in the field today that might be approached with the leg extensor include: 1) Determining the cellular mechanisms that underlie synaptic depression within high-output terminals (Is the depression due to a reduction of Ca2+ entry, lack of a competent readily releasable vesicle (RRV) pool, and/or altered postsynaptic receptivity?) 2) Determining the mechanistic role of 5-HT when applied after the induction of synaptic depression to promote a faster recovery, and 3) Determining whether the shapes of the quantal currents that arise from stimulating phasic terminals are being altered during the induction of depression to address pre- and post-synaptic components of synaptic depression.

This NMJ is important to ongoing research and future investigators because it enables us to obtain pertinent information that addresses the underling mechanisms of synaptic performance as measured directly at the release sites. Current research in this area is providing information about the modulation of synaptic depression by 5-HT and the dynamics of the vesicle pools. Such topics pertain to the fundamental basics of synaptic transmission relevant to all neural systems.

## Disclosures

No conflicts of interest declared.

## References

[B0] Atwood HL (1976). Organization and synaptic physiology of crustacean neuromuscular systems. Prog. Neurobiol.

[B1] Atwood HL, Cooper RL (1996). Synaptic diversity and differentiation: Crustacean neuromuscular junctions. Invertebrate Neuroscience.

[B2] Bradacs H, Cooper RL, Msghina M, Atwood HL (1997). Differential physiology and morphology of phasic and tonic motor axons in a crayfish limb extensor muscle. J. Exp. Biol.

[B3] Cooper RL, Stewart BA, Wojtowicz JM, Wang S, Atwood HL (1995). Quantal measurement and analysis methods compared for crayfish and Drosophila neuromuscular junctions, and rat hippocampus. J. Neurosci Methods.

[B4] Cooper RL, Donmezer A, Shearer J (2003). Intrinsic differences in sensitivity to 5-HT between high- and low-output terminals innervating the same target. Neuroscience Research.

[B5] Cooper AS, Cooper RL (2009). Historical view and demonstration of physiology at the NMJ at the crayfish opener muscle. J Vis Exp.

[B6] Del Castillo J, Katz B (1954). Quantal components of the end-plate potential. J. Physiol.

[B7] Desai-Shah M, Viele K, Sparks G, Nadolski J, Hayden B, Srinivasan VK, Cooper RL (2008). Assessment of synaptic function during short-term facilitation in motor nerve terminals in the crayfish. The Open Neuroscience Journal.

[B8] Desai-Shah M, Cooper RL (2009). Different mechanisms of Ca2+ regulation that influence synaptic transmission: Comparison between crayfish and Drosophila neuromuscular junctions. SYNAPSE.

[B9] Dudel J (1981). The effect of reduced calcium on quantal unit at the crayfish neuromuscular junction. Pfl gers Arch.

[B10] Griffis B, Moffett S, Cooper RL (2001). Muscle phenotype remains unaltered after limb autotomy and unloading. J. Exp. Zool.

[B11] Harata N, Pyle JL, Aravanis AM, Mozhayeva M, Kavalai ET, Tsien RW (2001). Limited numbers of recycling vesicles in small CNS nerve terminals: implications for neural signaling and vesicular cycling. Trends in Neurosci.

[B12] Jeromin A, Shayan AJ, Msghina M, Roder J, Atwood HL (1999). Crustacean frequenins: molecular cloning and differential localization at neuromuscular junctions. J. Neurobiol.

[B13] Johnstone AFM, Kellie S, Cooper RL (2008). Presynaptic depression in phasic motor nerve terminals and influence of 5-HT on docked vesicles. The Open Neuroscience Journal.

[B14] Katz B, Kuffler SW (1946). Excitation of the nerve-muscle system in Crustacea. Proc. Roy. Soc. B.

[B15] Kennedy D, Takeda K (1965). Reflex control of abdominal flexor muscles in crayfish. I. The twitch system. J. Exp. Biol.

[B16] Kennedy D, Takeda K (1965). Reflex control of abdominal flexor muscles in crayfish. II. The tonic system. J. Exp. Biol.

[B17] King MJR, Atwood HL, Govind CK (1996). Structural features of crayfish phasic and tonic neuromuscular junctions. J. Comp. Neurol.

[B18] LaFramboise W, Griffis B, Bonner P, Warren W, Scalise D, Guthrie RD, Cooper RL (2000). Muscle type specific myosin isoforms in crustacean muscles. J. Exp. Zool.

[B19] Logsdon S, Johnstone AFM, Viele K, Cooper RL (2006). Regulation of synaptic vesicles pools within motor nerve terminals during short-term facilitation and neuromodulation. J. Applied Physiol.

[B20] Lucas K (1907). The analysis of complex excitable tissues by their response to electric currents of short duration. J. Physiol.

[B21] Lucas K (1917). On summation of propagated disturbances in the claw of Astacus and on the double neuromuscular system of the abductor. J. Physiol.

[B22] Magrassi L, Purves D, Lichtman JW (1987). Fluorescent probes that stain living nerve terminals. J. Neurosci.

[B23] Millar AG, Bradacs H, Charlton MP, Atwood HL (2002). Inverse relationship between release probability and readily releasable vesicles in depressing and facilitating synapses. J. Neurosci.

[B24] Millar AG, Zucker RS, Ellis-Davies CG, Charlton MP, Atwood HL (2005). Calcium sensitivity of neurotransmitter release differs at phasic and tonic synapses. J. Neurosci.

[B25] Msghina M, Govind CK, Atwood HL (1998). Synaptic structure and transmitter release in crustacean phasic and tonic motor neurons. J. Neurosci.

[B26] Msghina M, Millar AG, Charlton MP, Govind CK, Atwood HL (1999). Calcium entry related to active zones and differences in transmitter release at phasic and tonic synapses. J. Neurosci.

[B27] Mykles DL, Medler SA, Koenders A, Cooper RL (2002). Myofibrillar protein isoform expression is correlated with synaptic efficacy in slow fibres of the claw and leg opener muscles of crayfish and.

[B28] Sparks G, Cooper RL (2004). 5-HT offsets homeostasis of synaptic transmission during short-term facilitation. J. Applied Physiol.

[B29] Sohn J, Mykles DL, Cooper RL (2004). The anatomical, physiological and biochemical characterization of muscles associated with the articulating membrane in the dorsal surface of the crayfish abdomen. J. Exp. Zool.

[B30] Tsien RW, Harata NC, Choi SWPyle, Aravanis AM, AM GChen (2001). Vesicles, quanta and synaptic plasticity. Molecular Mechanisms of synaptic function.

[B31] Van Harreveld A (1936). A physiological solution for freshwater crustaceans. Proc. Soc. Exp. Biol. Med.

[B32] Van Harreveld A, Wiersma CAG (1936). The triple innervation of the crayfish muscle. Proc. Natl. Acad. Sci. USA.

[B33] Wiersma CAG, Van Harreveld A (1938). The influence of the frequency of stimulation on the slow and fast contraction in crustacean muscle. Physiol. Zool.

[B34] Wojtowicz JM, Smith BR, Atwood HL (1991). Activity-dependent recruitment of silent synapses. Ann. N.Y. Acad. Sci.

